# Suppression of FGF5 and FGF18 Expression by Cholesterol-Modified siRNAs Promotes Hair Growth in Mice

**DOI:** 10.3389/fphar.2021.666860

**Published:** 2021-07-07

**Authors:** Jungang Zhao, Haojie Lin, Lusheng Wang, Keke Guo, Rongrong Jing, Xuenan Li, Yu Chen, Zhenlin Hu, Shuang Gao, Nuo Xu

**Affiliations:** ^1^ College of Life and Environmental Sciences, Wenzhou University, Wenzhou, China; ^2^ School of Pharmaceutical Sciences, Wenzhou Medical University, Wenzhou, China

**Keywords:** fibroblast growth factors, FGF5, FGF18, hair follicle cycle, cholesterol-modified siRNA

## Abstract

FGF5 and FGF18 are key factors in the regulation of the hair follicle cycle. FGF5 is overexpressed during the late anagen phase and serves as a crucial regulatory factor that promotes the anagen-to-catagen transition in the hair follicle cycle. FGF18, which is overexpressed during the telogen phase, mainly regulates the hair follicle cycle by maintaining the telogen phase and inhibiting the entry of hair follicles into the anagen phase. The inhibition of FGF5 may prolong the anagen phase, whereas the inhibition of FGF18 may promote the transition of the hair follicles from the telogen phase to the anagen phase. In the present study, we used siRNA to suppress FGF5 or FGF18 expression as a way to inhibit the activity of these genes. Using qPCR, we showed that FGF5-targeting siRNA modified by cholesterol was more effective than the same siRNA bound to a cell-penetrating peptide at suppressing the expression of FGF5 both *in vitro* and *in vivo*. We then investigated the effects of the cholesterol-modified siRNA targeting either FGF5 or FGF18 on the hair follicle cycle in a depilated area of the skin on the back of mice. The cholesterol-modified siRNA, delivered by intradermal injection, effectively regulated the hair follicle cycle by inhibiting the expression of FGF5 and FGF18. More specifically, intradermal injection of a cholesterol-modified FGF5-targeted siRNA effectively prolonged the anagen phase of the hair follicles, whereas intradermal injection of the cholesterol-modified FGF18-targeted siRNA led to the mobilization of telogen follicles to enter the anagen phase earlier. The inhibitory effect of the cholesterol-modified FGF18-targeted siRNA on FGF18 expression was also evaluated for a topically applied siRNA. Topical application of a cream containing the cholesterol-modified FGF18-targeted siRNA on a depilated area of the skin of the back of mice revealed comparable inhibition of FGF18 expression with that observed for the same siRNA delivered by intradermal injection. These findings suggested that alopecia could be prevented and hair regrowth could be restored either through the intradermal injection of cholesterol-modified siRNA targeting FGF5 or FGF18 or the topical application of FGF18 siRNA.

## Introduction

Hair follicles, which are major accessory organs of the skin, control the cyclic growth of hairs (Chen et al., 2013). They can regenerate and undergo cyclical changes in growth. The growth cycle of hair follicles supports the self-renewal of hair follicles and regulates hair growth and shedding. Hair follicles consist of an upper permanent portion and a lower cyclical portion. Based on the changes that occur in the cyclical portion, the hair follicle cycle is divided into the anagen, catagen, and telogen phases (Higgins et al., 2014). Abnormalities in the hair follicle cycle are a key mechanism in the pathogenesis of alopecia. The anagen phase in normal scalp hair follicles lasts for 2–6 years, but this may be shortened to several months to a year for the hair follicles on the crown in androgenetic alopecia, which is the most common form of alopecia (Ben David-Naim et al., 2017). Continuous shortening of the anagen phase leads to progressive hair follicle miniaturization, thus prolonging the telogen phase. Ultimately, the hair follicles become arrested at the telogen phase, which results in alopecia. Shortening of the anagen phase is caused by premature termination of the anagen phase followed by premature entry into the catagen phase. Therefore, the key to preventing alopecia is to prevent the progressive shortening and premature termination of the anagen phase, and the key to restoring hair growth is the mobilization of telogen follicles to enter the anagen phase.

The mechanisms involved in the regulation of hair growth are extremely complex. Research in recent years has revealed that many growth factors, including fibroblast growth factors (FGFs), participate in the regulation of the hair follicle cycle (Kawano et al., 2005). FGFs, a large 23-member family of growth factors, mediate FGF signal transduction by binding to FGF receptors (FGFRs) and forming ternary complexes with heparan sulfate proteoglycans (HSPGs) or members of the Klotho family. Various members of the FGF family participate in the regulation of the hair follicle cycle, with FGF1, FGF2, FGF7, and FGF10 being highly expressed and promoting hair growth during the anagen phase. Notably, FGF5, which is an inhibitor of hair growth, is highly expressed during the late anagen phase. It is currently regarded as the most effective factor for promoting the anagen-to-catagen transition of hair follicles (Kehler et al., 2007; He et al., 2016; Jin and Zhang 2018). FGF5 was first discovered in angora mice, which carry a spontaneous mutation in the FGF5 gene. Angora mice were characterized by a prolonged anagen phase for the hair follicles, an abnormal increase in the proportion of anagen hair follicles, and a 50% longer hair coat length compared with that of the wild-type mice. Mutations in the FGF5 gene form the genetic basis for familial trichomegaly, which is manifested as increased hair growth and abnormally long and thick eyelashes for the carriers of the mutated FGF5 gene. FGF5-deficient or knockout animals (including mice, sheep, cats, dogs and guinea pigs) also have abnormally thick and long hair (Yu et al., 2018). A previous study has shown that an FGF5-derived decapeptide that possesses FGF5-antagonizing activity can promote hair growth in mice (Ito et al., 2003). Therefore, inhibiting the expression of the FGF5 gene or antagonizing its activity will prevent the premature termination of the anagen phase of the hair follicles, thereby preventing alopecia.

FGF18 is overexpressed during the telogen phase to maintain the quiescent stage of the hair follicles and to facilitate their entry into the anagen phase. When the hair follicles are in the late anagen phase, subcutaneous injection of FGF18 into skin of mice can effectively inhibit the growth of hair follicles (Blanpain et al., 2004). When the FGF18 gene was conditionally knocked out in keratin 5-positive epithelial cells in mice, the telogen phase was dramatically shortened, causing a strikingly rapid succession of hair cycles, demonstrating that the absence of FGF18 signaling significantly accelerates the start of anagen phase (Kimura-Ueki et al., 2012). These studies indicate that FGF18 is a key regulatory factor in the maintenance of the telogen phase in hair follicles and that inhibition of its expression or antagonism of its activity can promote the telogen-to-anagen transition, which will ultimately lead to hair regrowth.

Small interfering RNA (siRNA)-mediated inhibition of gene expression, which is characterized by its high specificity and high efficiency, has become an important technique for studying gene function and for drug target discovery research. Dozens of siRNA drugs are currently being studied in clinical trials for the treatment of a wide variety of diseases such as malignant tumors, viral infections, diabetic macular edema, asthma, eye diseases, and skin diseases (Sajid and Moazzam 2020; Saw and Song 2020). However, two major barriers exist in the local application of siRNA to the skin, namely the inability of siRNA to penetrate the stratum corneum barrier and poor efficiency in the uptake of the siRNA by the skin cells. The stratum corneum, which is the outermost layer of the skin, has a thickness of 10–20 µm and is composed of multiple layers of corneocytes (i.e., keratinocytes in their last stage of differentiation). Hydrophilic compounds with a molecular weight of >500 Da are therefore unable to penetrate the stratum corneum freely (Steven and Steinert, 1994). As siRNA molecules generally have a molecular weight of >13,000 Da, a negative charge, and a high degree of hydrophilicity, they are incapable of penetrating the stratum corneum barrier in the absence of an assisted delivery method. Assuming that the stratum corneum barrier could be overcome, the lipid bilayer structure of the cell membrane still makes it difficult for the negatively charged siRNAs to enter the skin cell. Previous research has indicated that transfection agents such as cationic liposomes must be used during *in vitro* cell experiments to allow the siRNA to enter the cell and exert its intended effects (Hickerson et al., 2011). Studies have found that the covalent conjugation of a cholesterol molecule to the 3′ or 5′ end of siRNA greatly enhances its *in vivo* stability and increases its hydrophobicity and ability to penetrate the membrane (Soutschek et al., 2004; Chong et al., 2013). The cholesterol-modified siRNA, which has a high transfection efficiency is safe and can act as a self-delivering siRNA that can effectively interfere with the expression of target genes. One recent study has revealed that topical application of a cream containing a cholesterol-modified siRNA can efficiently deliver the siRNA through the stratum corneum barrier to allow its subsequent entry into the epithelial cells, thereby effectively inhibiting the expression of the target gene in the epithelial cells to <50% without producing any obvious adverse effect (Wolfrum et al., 2007). Such cholesterol-modified siRNA creams may potentially be developed into a simple, convenient, efficient, and safe RNA interference-based product for the treatment of skin diseases.

Cell-penetrating peptides (CPPs) are short peptides that are capable of carrying macromolecules such as proteins and nucleic acids across the cell membrane and into the cell (Beloor et al., 2015). The non-covalent interaction between CPPs and siRNAs can lead to the formation of stable nanocomposites (Falato et al., 2021). These nanocomposites have been demonstrated to have a good delivery efficiency in various cell lines (Nakase et al., 2013). Besides efficiently transporting the siRNAs into the cells, CPPs can also facilitate the penetration of siRNAs across the stratum corneum barrier (Uchida et al., 2011; Chen et al., 2014).

In the present study, FGF5 and FGF18, which are key factors that negatively regulate the hair follicle cycle, were selected for inhibition by targeted siRNAs and the effects of these siRNAs on the hair follicle growth cycle were examined. Different formulations of the siRNAs (cholesterol-conjugated or CPP-bound) were compared. In addition, delivery by intradermal injection and via topical application were also evaluated. The results of this study may provide a scientific basis for the development of novel hair loss prevention products suitable for topical application.

## Materials and Methods

### Preparation of siRNA Samples

Synthesis of the candidate siRNAs and the subsequent covalent modifications at the 5′end with cholesterol were performed by GenePharma (Shanghai, China). Three FGF5-targeting siRNAs were designed: 567–25/27, 635–25/27 and 565–25/27. The numbers 567, 653 and 565 indicate the start of the binding site in the target gene, while 25/27 indicates the length of the siRNA sequence. The sequences of the siRNAs are as follows: FGF5 siRNA-635–25/27-sense 5′-GCA​ACA​AAU​UUU​UAG​CGA​UGU​CAA​A-3′ and antisense 5′-UUU​GAC​AUC​GCU​AAA​AAU​UUG​UUG​CUG-3'; FGF5 siRNA-565–25/27-sense 5′-GCC​AGA​GAG​UUA​AGU​AUU​UUG​GAA​A-3′ and antisense 5′-UUU​CCA​AAA​UAC​UUA​ACU​CUC​UGG​CUU-3'; FGF5 siRNA-567–25/27-sense5′-CAGUGUGUUAAGUAUUUUGGAAAUA-3′ and antisense 5′-UAU​UUC​CAA​AAU​ACU​UAA​CAC​ACU​GGC-3'; FGF5 siRNA-567–25/27-NC-sense 5′-GUU​GUA​AUC​UUG​AUG​AUA​AUG​AGU​A-3′ and antisense 5′-UAC​UCA​UUA​UCA​UCA​AGA​UUA​CAA​CGC-3'; FGF8 siRNA-502–25/27-sense 5′-GGA​GUG​CGU​GUU​CAU​UGA​GAA​GGU​U-3′ and antisense 5′-AAC​CUU​CUC​AAU​GAA​CAC​GCA​CUC​CUU-3'; FGF8 siRNA-502–25/27-NC-sense 5′-GUC​AUU​GAG​AAG​GUG​AGU​GCG​UGU​U-3′ and antisense 5′-AAC​ACG​CAC​UCA​CCU​UCU​CAA​UGA​CUU-3'.

For intradermal injection, the cholesterol-modified siRNA and the siRNA containing a negative control sequence were each diluted 20 μM. To prepare the siRNA cream for topical application, 330 μg cholesterol-modified siRNA was first dissolved in 0.25 g of azone and 2.0 g of 2-ethylhexyl palmitate to form an oil phase. Next, 1.0 g of propylene glycol and 0.25 g of benzethonium chloride were dissolved in water to form an aqueous phase. After that, an emulsifier was added to the oil phase and stirred slowly to achieve a uniform mixture. Finally, the aqueous phase was slowly added to the oil phase in a batch-wize manner and stirred to obtain a uniform mixture, which was then stored in a separate container.

### Cell Line and Cell Culture

NIH-3T3 cells (mouse embryonic cell line) were obtained from the Cell Bank of the Chinese Academy of Sciences (Shanghai, China) and cultured in Dulbecco’s modified Eagle’s medium (DMEM) containing 10% fetal bovine serum (FBS) (Gibco, Life Technologies Corporation, NY, United States) and 1% penicillin/streptomycin at 37°C in a 5% CO_2_ incubator.

### Animal and Housing

Healthy C57BL/6 mice (female, 18–22 g) were purchased from Shanghai SLAC Laboratory Animal Co., Ltd. (Shanghai, China). Animal care was performed in accordance with international ethical guidelines and the National Institutes of Health Guide for the Care and Use of Laboratory Animals. All animals were housed under controlled temperature (20 ± 2°C) with a 12 h light/12 h dark cycle.

### Transfection of NIH/3T3 Cells With FGF5/FGF18 Plasmid and Liposome-Encapsulated, Cholesterol-Modified, and CPP-Bound siRNAs

NIH/3T3 cells that overexpressed either FGF5 or FGF18 were prepared by inoculating the cells onto 6-well plates at a density of 1×10^5^ cells/well in 2 ml complete medium containing serum and penicillin/streptomycin. When the cells reached 80% confluence, the medium was replaced with 2 ml fresh medium without penicillin/streptomycin. Next,1.6 μg of FGF5 or FGF18 expression plasmid DNA and 4 μL lipofectamine 2000 were mixed in 200 μL DMEM medium without serum and penicillin/streptomycin and left to stand for 5 min. After that, the sample was added to the cells and the plate was then incubated at 37°C for 6 h in a 5% CO_2_ incubator. The culture medium was replaced with fresh medium and the cells were transfected with siRNA as described below. NIH/3T3 cells that did not overexpress FGF5 or FGF18 were also prepared in the same way, but the cells were instead transfected with the empty plasmid DNA.

To prepare the Lipofectamine 2000-mediated siRNA, 200 pmol siRNA was first diluted in 100 μL of serum-free medium without penicillin/streptomycin, while 4 μL of Lipofectamine 2000 was added to a separate 100 μL of serum-free medium, and the two samples were each stirred gently, left to stand for 5 min, and then thoroughly mixed and left to stand for another 20 min. To prepare the cholesterol-modified siRNA, 200 pmol of the cholesterol-modified siRNA was diluted and gently mixed with 200 μL of serum-free medium without penicillin/streptomycin and left to stand for 5 min. To prepare the CPP-bound siRNA, 200 pmol of siRNA was diluted in 100 ml of culture medium without serum and penicillin/streptomycin, while 600 pmol of CPP stearyl-R8 was diluted in a separate 100 ml of serum-free medium without penicillin/streptomycin. Each sample was gently stirred, left to stand for 5 min and then thoroughly mixed and left to stand for a further 20 min. To perform the transfection, each of the siRNA preparation was added to separate wells in a 6-well plate containing the NIH/3T3 cells, and the plate was then incubated at 37°C for 24 h in a 5% CO_2_ incubator.

### Administration of FGF5 siRNA to the Skin and Collection of Tissue Samples

Seven-week-old female C57BL/6 mice were anesthetized using a small animal inhalation anesthesia machine. Depilation wax was prepared by mixing rosin and paraffin wax in a 1:1 ratio. The mixture was melted by heating, and then evenly applied to the back of each animal. After complete solidification, the depilation wax was slowly peeled off using a pair of tweezers to completely remove the hairs, and the date of depilation was recorded as Day 0. the purpose of using depilatory wax to completely remove hair is to stimulate hair follicles to enter the growth phase on the next day. The back of each animal was divided into two zones, left and right zones. The left zone was injected with the siRNA containing a negative control sequence, while the right zone was injected with the cholesterol-modified siRNA. For each injection, 50 μL of each siRNA sample was intradermally injected at five points in the appropriate skin region for even distribution of the siRNA. For the mice in this part of the experiment, we observed for 29 days. The siRNA was injected on days 14, 17, and 20 following depilation. From Day 15 to Day 29, skin specimens were collected from the regions of siRNA injection once every 2 days. The specimens were obtained from three mice at each collection time point. Each skin tissue specimen was divided into three portions, with one portion fixed in 4% paraformaldehyde solution for histochemical staining and microscopic observation while the remaining portions were stored in two separate 1.5 ml EP tubes at −80°C for quantitative polymerase chain reaction (qPCR) and western blot assays.

### Administration of FGF18 siRNA and Collection of Skin Tissue Samples

Seven-week-old female C57BL/6 mice were anesthetized using a small animal inhalation anesthesia machine. Surface hairs on the skin of the back of each animal were removed using an electric shaver. Subsequently, depilation wax was applied to the shaved region, left on for 10 min, and removed by gentle rubbing with a piece of gauze to achieve complete surface hair removal. The purpose of using depilatory wax to surface hair removal is for topical application and to observe the black spots in the skin of the depilated regions. The back-depilated mice were randomly divided into two groups, which were separately administered siRNAs by intradermal injections or with topical application to compare the *in vivo* pharmacological effects of the two drug administration methods. The back of each animal was divided into two zones, left and right zones. The left zone was injected with the siRNA containing a negative control sequence or smeared with a cream containing the same siRNA, whereas the right zone was injected with the cholesterol-modified FGF18-targeting siRNA or smeared with a cream containing the same siRNA. Topical application was commenced on Day 1 following depilation and performed once daily for 15 consecutive days. For injection, 50 μL of the siRNA was intradermally injected at five points in the corresponding skin region. For the mice in this part of the experiment, we observed for 15 days. The siRNA was injected on Days 1, 4, 7 and 10 following depilation. From Day 3 to Day 15, hair growth in the siRNA-administered regions of the skin was recorded by photographing with a digital camera and a hair follicle detector once every 3 days. Skin tissue specimens were also collected at the time of recording. Each skin tissue specimen was divided into three portions as treated as described in Administration of FGF5 siRNA to the skin and collection of tissue samples.

### ELISA

NIH-3T3 cultures, both plasmids or siRNAs-treated, were centrifuged to obtain the culture supernatants, and the extent of FGF5 secretion in the supernatants was then measured with an ELISA kit (HUAMEI, Wuhan, China) according to the manufacturer’s instruction.

### Reverse Transcription-Quantitative Polymerase Chain Reaction

Total RNA was extracted from skin specimens Using TRIzol® Reagent (Thermo Fisher Scientific, Waltham, MA, United States). And total RNA was extracted from cultured cells using an RNA isolation kit (Biomiga, San Diego, CA, United States) according to the manufacturer’s instruction. The extracted RNA was used as a template for reverse transcription performed with the PrimeScript RT reagent Kit (Takara, Dalian, China). Reverse transcription was carried out on an LC96 system (Roche, Basel, Switzerland) using the SYBR Green Master Mix (Applied Biosystems, Foster City, CA, United States). The following primers used: FGF5-forward, 5′-AAGTCAATGGCTCCCACGAAGC-3′and reverse 5′-CCG​TAA​ATT​TGG​CAC​TTG​CAT​GG-3'; FGF18-forward, 5′-GGAGCAGGTGACCTTTGATGAG-3′and reverse 5′-GAG​AGG​TGC​CAG​TTG​ATG​ATG​G-3'; GAPDH-forward5′-AGAAGGTGGTGAAGCAGGCATC-3′and reverse-5′-CGAAGGTGGAAGAGTGGGAGTTG-3'. The relative level of FGF5 or FGF18 mRNA was calculated using the comparative Ct method with GAPDH mRNA as an endogenous control. All data were normalized to that of the non-treated control.

### Western Blot

Extracts of the skin tissue or NIH-3T3 cells were prepared and then centrifuged at 12,000 × g to precipitate the insoluble materials. The protein concentration in the supernatant was determined and samples containing equal amounts (25/40 μg) of protein were separated by SDS-PAGE using 12% gel. The protein in the gel was then transferred to polyvinylidene difluoride (PVDF) membrane and blocked with 5% skim milk for 1 h followed by incubation with rabbit anti-FGF5 (Proteintech, Chicago, IL, United States), anti-FGF18 (Proteintech, Chicago, IL, United States), or anti-tubulin (Cell Signaling Technology, Beverly, MA, United States) for overnight at 4°C. After washing the blot was incubated with goat anti-rabbit antibody (Cell Signaling Technology, Beverly, MA). The blot was visualized with a chemiluminescence substrate (Pierce, Rockford, IL, United States) and images of the blot were captured with an Amersham Imager (GE Healthcare Biosciences, Pittsburgh, PA, United States) and analyzed using the ImageJ software (U. S. National Institutes of Health, Bethesda, Maryland, United States).

### Histological Examination

The skin tissue was soaked in 4% paraformaldehyde and stored in a 4°C refrigerator for 24–48 h. After that, it was dehydrated in ethanol solutions containing increasing concentrations of ethanol, and then embedded in paraffin, and subsequently sliced into 10-mm thick sections. The sections were stained with hematoxylin and eosin (HE) solution and observed under a Leica Microsystem (Wetzlar, Germany). To evaluate the expression of FGF5 and FGF18 in the hair follicles by immunohistochemistry, the sections were incubated in 3% H_2_O_2_ for 25 min and then blocked with 3% BSA for 4 h. They were then incubated with anti-FGF5 antibody (1: 500 dilution; Proteintech, Wuhan, China) or anti-FGF18 antibody (1:500 dilution; Proteintech, Wuhan, China) overnight at 4°C. After three washes with PBS, the sections were incubated with horseradish peroxidase-conjugated secondary antibodies for 4 h at 37°C and then with 3,3-diaminobenzidine (DAB) for about 3 min. All stained sections were examined with a DM3000 microscope (Leica, Wetzlar, Germany) to determine the histological changes.

### Statistical Analysis

All statistical analyses were performed with GraphPad Prism 6.0 software. The statistical differences between treatment groups and control were determined using one-way analysis of variance (ANOVA). All data were expressed as mean ± SD, and statistical significance was considered at the *p* < 0.05 level.

## Results

### Effects of Different Delivery Methods for FGF5-Targeted siRNAs at the Cellular and Tissue Levels

**FIGURE 1:**
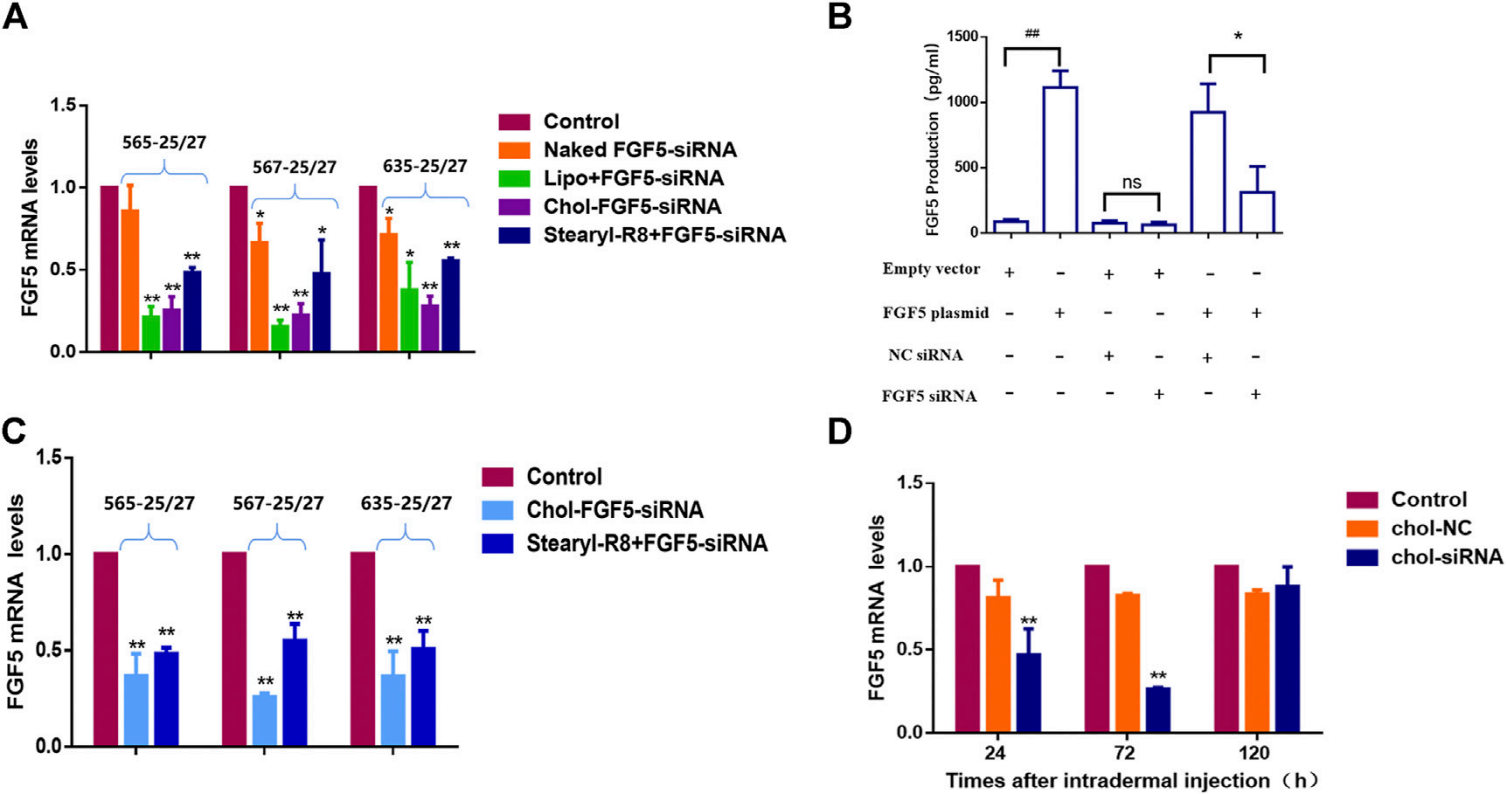
Effects of different FGF5-targeting siRNA delivery methods on the expression of FGF5 **(A)** Changes in FGF5 mRNA level in NIH-3T3 cells overexpressing the FGF5 gene that were transfected with different preparations of FGF5-targeting siRNA as determined by qPCR. NIH-3T3 cells were incubated without (control) or with naked FGF5 siRNA, lipo + FGF5 siRNA, chol-FGF5-siRNA or Stearyl-R8+FGF5-siRNA for 24 h and the levels of FGF5 mRNA in these cells were measured by qPCR. Data are mean ± SD from three experiments. *p < 0.05, **p < 0.01 vs. control **(B)** Effect of FGF5 siRNA on the expression of FGF5 in NIH-3T3 cells. NIH-3T3 cells were transfected with an empty vector or vector containing the FGF5 gene and the level of FGF5 secreted by the cells was then measured by ELISA following transfection with the cholesterol-modified negative control siRNA (NC siRNA) or cholesterol-modified FGF5 siRNA. Data are mean ± SD from three experiments. ^##^ *p* < 0.05, **p < 0.01 **(C)** Effect of FGF5 siRNA on FGF5 mRNA level in mouse skin. The skin of the animal was either not treated with siRNA (control) or treated with Chol-FGF5-siRNA or the Stearyl-R8 bound FGF5-siRNA by intradermal injections, and the level of FGF5 mRNA in the skin was measured by qPCR after 24 h. Data are mean ± SD (*n* = 3). **p < 0.01 vs. control **(D)** Changes in FGF5 mRNA level in mouse skin over time following the administration of FGF5 siRNA. Chol-FGF5-siRNA or Chol-NC-siRNA was administered to the skin of mice by intradermal injection, and the mRNA level of FGF5 in the skin was measured after 24, 72, 120 h, respectively, using qPCR. Data are mean ± SD (*n* = 3). **p < 0.01 vs. control.

Three candidates of FGF5-targeting siRNAs were tested and all were found to effectively inhibit the expression of FGF5 as evaluated using FGF5-overexpressing NIH/3T3 cells. Subsequently, each siRNA was separately subjected to liposome (Lipofectamine 2000) encapsulation, binding with CPP (stearyl-R8) and cholesterol modification. Compared with the naked siRNA, the liposome-encapsulated, CPP-bound and cholesterol-modified siRNAs all caused a significant decrease in FGF5 mRNA level, indicating that all three delivery methods allowed the siRNA to enter the cells to inhibit the expression of FGF5. However, the cholesterol-modified siRNA exerted a stronger inhibition than the CPP-bound siRNAs (Figure 1A), and 567–25/27 was the most potent. As FGF5 is an secretory protein, we used ELISA to detect FGF5 produced by NIH-3T3 cells. Compared with the cells transfected with the empty vector, those transfected with FGF5 containing plasmid produced significantly more FGF5. However, the amount of FGF5 produced by the cells that did not overexpress FGF5 did not change significantly when they were also transfected with the cholesterol-modified negative control siRNA or FGF5 siRNA (567–25/27) (Figure 1B). In contrast, cells that overexpressed FGF5 displayed a significant decrease in FGF5 production when they were transfected with the cholesterol-modified FGF5 siRNA (567–25/27), consistent with the qPCR result. Although Lipofectamine 2000 is commonly used in a laboratory for the transfection of cells with siRNAs, it cannot be used for *in vivo* experimentation because of its cytotoxicity. The efficiency of FGF5 knockdown by the cholesterol-modified and CPP-bound siRNAs in the mice skin was then determined. Both cholesterol-modified and CPP-bound siRNAs effectively reduced the level of FGF5 mRNA compared with the negative control siRNA (Figure 1C), with the cholesterol-modified siRNA 567–25/27 exerting a strongest effect. Thus, 567–25/27 was chosen for subsequent investigation of the mouse hair follicle cycle. To determine the duration of the inhibition of FGF5 expression by siRNA in mice, we measured the level of FGF5 mRNA at 24, 72, and 120 h after intradermal injection of the cholesterol-modified 567–25/27. The level of FGF5 mRNA dropped by as much as 55.37 ± 7.64% 24 h after injection, and 73.62 ± 0.69% 72 h after injection compared with the level of FGF5 mRNA in the skin injected with the negative control siRNA. The suppressive effects of siRNA had almost completely disappeared 120 h after injection (Figure 1D), indicating that the intradermally injected cholesterol-modified siRNA exerted a continuous inhibitory effect on the target gene for up to 72 h post-injection. Therefore, the cholesterol-modified 567–25/27 was injected intradermally once every 72 h to further investigate the effect of inhibition of FGF5 on the hair follicle cycle in mouse skin.

### Effects of Chol-FGF5-siRNA on FGF5 Expression and Hair Follicle Cycle Status in Mouse Skin

**FIGURE 2:**
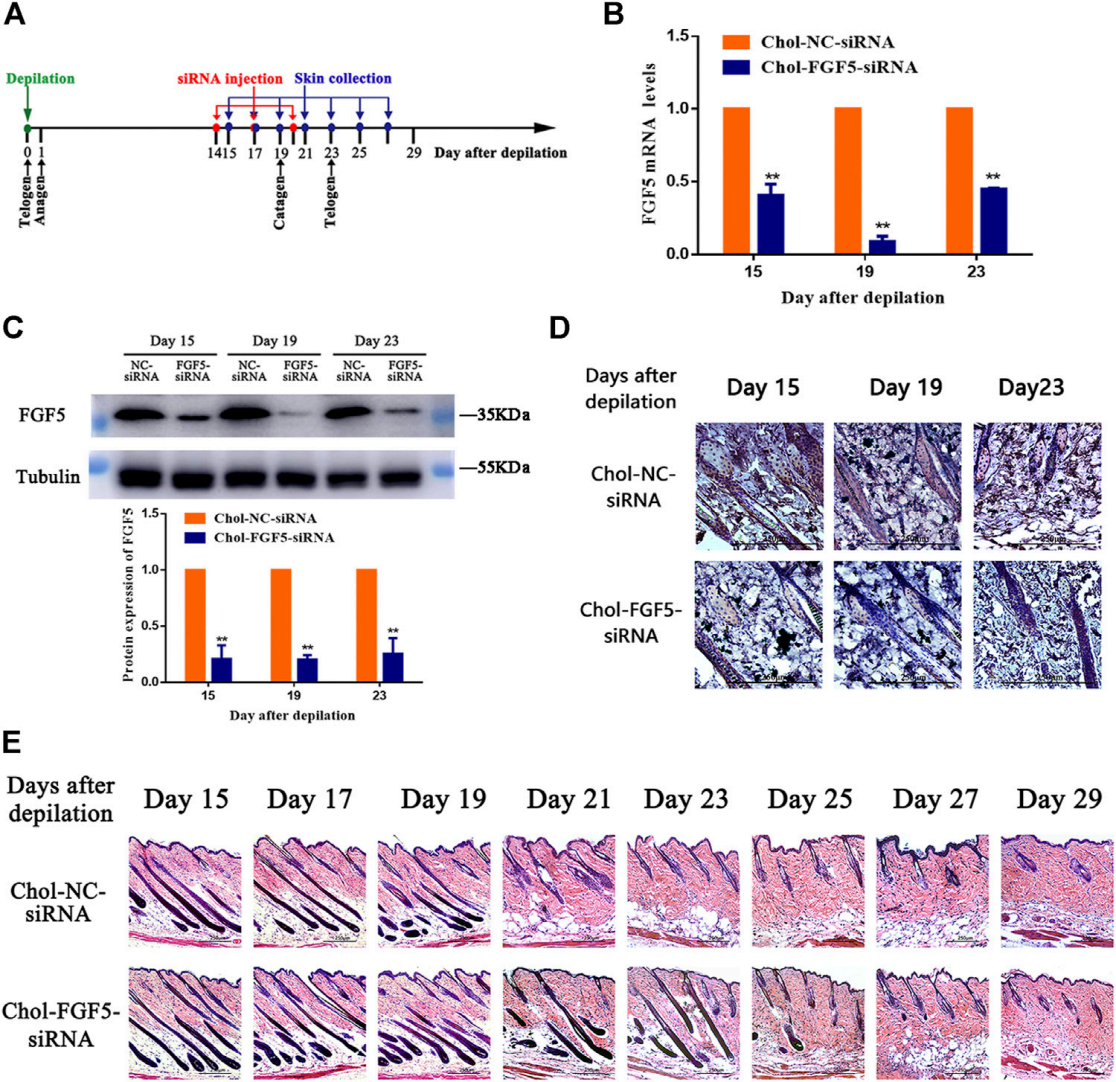
Effects of Chol-FGF5-siRNA on the expression of FGF5 and hair follicle cycle in mouse skin **(A)** Diagram depicting the timeline in the establishment of the model used for the administration of Chol-siRNA and collection of skin samples. **(B)** Changes in FGF5 mRNA expression in mouse skin as determined by qPCR. Chol-FGF5-siRNA or Chol-NC-siRNA was delivered to the mouse skin by intradermal injection 14 days after depilation, and the mRNA level of FGF5 in the skin was measured by qPCR at the indicated times **(C)** Changes in FGF5 protein expression in mouse skin as determined by western blot. Chol-FGF5-siRNA or Chol-NC-siRNA was delivered to the mouse skin by intradermal injection 14 days after depilation, and the expression of FGF5 protein in the skin was measured by western blot at the indicated times. The plot below the blot shows the quantitation of the protein in the blot as determined by gray analysis. **(D)** Immunohistochemical staining of the skin tissue **(E)** HE staining of skin tissue showing the different phases of the hair follicle cycle. All graphical data are mean ± SD (*n* = 3). **p < 0.01 vs. Chol-NC-siRNA.

Figure 2A shows the timeline for the depilation of mice skin, administration of siRNA and collection of skin samples for further analysis. According to the qPCR result, the level of FGF5 mRNA in the skin tissue of mice injected with the cholesterol-modified FGF5 siRNA was reduced by 59.32 ± 4.37%, 91.08 ± 2.13%, and 55.14 ± 0.48% on days 15, 19, and 23 respectively, compared with mice intradermally injected with the cholesterol-modified negative control siRNA. Consistently, the level of FGF5 protein in the skin injected with the FGF5 siRNA was also significantly reduced on days 15, 19 and 23 compared the negative control siRNA (Figure 2C). Immunohistochemical analysis of the skin tissue further confirmed that injection with FGF5 siRNA effectively inhibited the expression of FGF5 in the skin (Figure 2D). Histochemical staining of the hair follicles in both groups revealed thick hair bulbs located at the dermal-subcutaneous junction on days 15, 17 and 19, indicating that the hair follicles were in the anagen phase, with the presence of vigorous hair growth. From day 21 onwards, the hair follicles of the negative control group started to diminish in size while the hair bulbs at the base of the follicles shrank and migrated upwards, indicating that these hair bulbs were undergoing the anagen-to-catagen transition. By day 23, the hair follicles were in the catagen phase. However, in the FGF5 siRNA group, the hair follicles began to exhibit signs of the anagen-to-catagen transition (i.e., shrinkage and upward migration) only from day 23 onwards (Figure 2E). The result indicated that intradermal injection of the cholesterol-modified FGF5 siRNA (567–25/27) into mouse skin could effectively inhibit the expression of FGF5, prolonging the anagen phase of hair follicles by two days, and delaying the entry of hair follicles into the catagen phase.

### Effects of Intradermal Injection of a Cholesterol-Modified FGF18-Targeted siRNA in Mouse Skin Tissue

**FIGURE 3:**
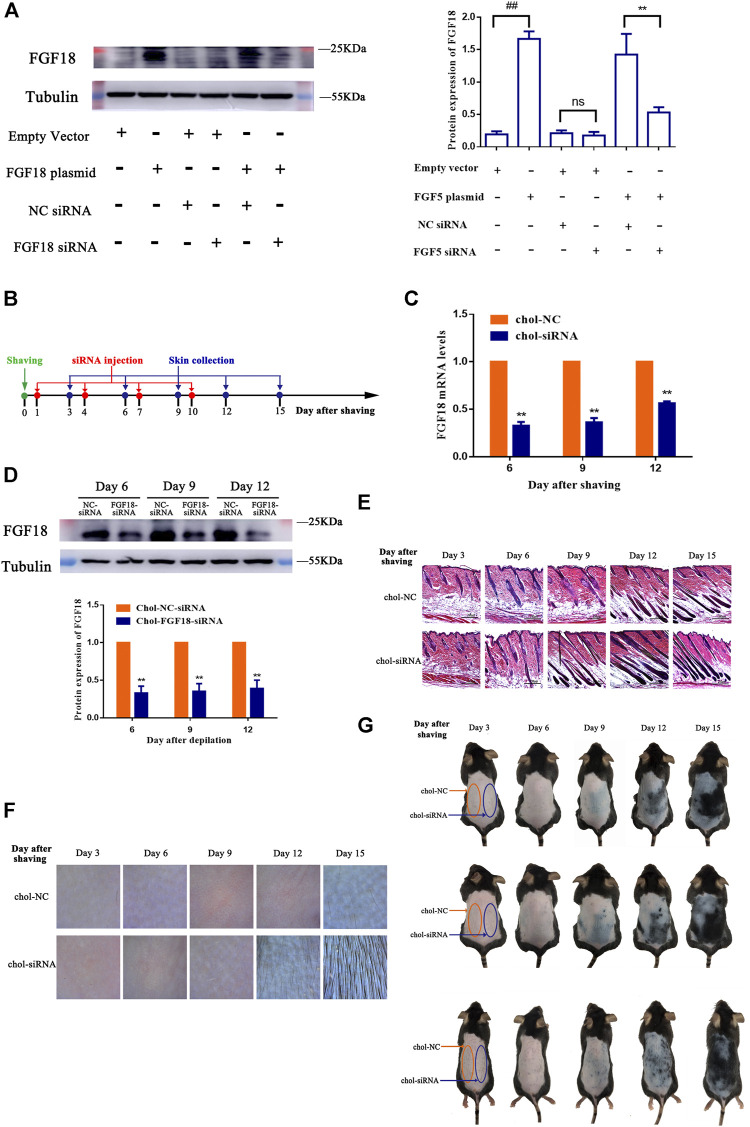
Effect of FGF8 siRNA on the expression of FGF8 in mouse skin **(A)** Effect of FGF18 siRNA on the expression of FGF18 in NIH-3T3 cells. NIH-3T3 cells were transfected with an empty vector or vector containing the FGF18 gene and the level of FGF18 secreted by the cells was then measured by western blot following transfection with the cholesterol-modified negative control siRNA (NC siRNA) or cholesterol-modified FGF18 siRNA. The plot shows the corresponding western blot result. Data are mean ± SD (*n* = 3). **p < 0.01, ^##^ *p* < 0.01 **(B)** Diagram depicting the timeline in the establishment of the model used for the administration of Chol-FGF18-siRNA and collection of skin samples. Chol-FGF18-siRNA or Chol-NC-siRNA was delivered to the mouse skin by intradermal injection one day after depilation, and the mRNA level of FGF18 in the skin tissue was measured by PCR **(C)** whereas the protein level of FGF18 was measured by western blot **(D)** at the indicated times. The plot below the blot in **(D)** shows the quantitation of the protein bands in the blot as determined by grayscale. Data are mean ± SD (*n* = 3). **p < 0.01 vs. Chol-NC siRNA **(E)** HE staining of skin tissue showing the different phases of the hair follicle cycle. **(F)** Digital images showing the extent of hair growth on the skin as detected by a hair follicle detector at 200× magnification following injection of siRNA. **(G)** Digital images showing the back of the mice after injection with FGF18-targeted siRNA and NC-siRNA following depilation.

The effect of FGF18 siRNA on the expression of FGF18 was determined essentially following the same approach as that of FGF5. NIH-3T3 cells were either transfected with the empty vector or FGF18 containing plasmid without or with subsequent transfection with the cholesterol-modified negative control siRNA or FGF18 siRNA, and the level of FGF18 protein was then measured by western blot. Again, cells that transfected with the FGF18 containing plasmid showed significantly higher level of FGF18 protein than those transfected with the empty vector, confirming the overexpression of FGF18 in these cells (Figure 3A). Following the transfection with the negative control siRNA or FGF18 siRNA, the cells that did not overexpress FGF18 showed not significant change in FGF18 protein level, but the cells that overexpressed FGF18 displayed a significant decrease in FGF18 protein. This result confirmed that FGF18 siRNA could specifically interfere with the expression of FGF18 in the cells. Figure 3B shows the timeline for the depilation of mice skin, administration of FGF18 siRNA and collection of skin samples for further analysis to assess the impact on the hair follicles. The level of FGF18 mRNA in the skin tissue samples of mice given the FGF18 siRNA was significantly lower than in the mice given the negative control siRNA (Figure 3C). At the same time, western blot also revealed a significant decrease in the level of FGF18 protein in the skin injected with FGF18 siRNA compared with that in the skin injected with the negative control siRNA (Figure 3D). On day 9, the hair follicles in the group injected with the FGF18 siRNA exhibited hair growth and plump papillae that were located at the dermal-subcutaneous junction, indicating that the hair follicles were in the anagen phase. Similar growth was only achieved on day 12 in the hair follicles of the group injected with the negative control siRNA (Figure 3E), demonstrating FGF18 siRNA could result in earlier transition of the telogen-to-anagen phase for the hair follicles. On day 9, photographs taken through hair follicle detector and ordinary digital camera show photographs and a hair follicle detector showed the presence of black spots in FGF18 siRNA-administered zone. Obvious hair growth occurred by day 12 and thick hair appeared on day 15. In contrast, the zone of the skin that was injected with the negative control siRNA zone remained pink on day 9 and did not exhibit obvious hair growth on day 12. Hair growth finally occurred on day 15, but the regrown hair was relatively sparse (Figures 3F,G). Taken together, the results showed that following the intradermal injection of a cholesterol-modified FGF18 siRNA, there was an effective reduction in FGF18 gene expression in the skin tissue, the telogen-to-anagen transition was shorten by 3 days, with was the stimulation of hair growth.

### Effects of siRNA Application of a Cholesterol-Modified FGF18-Targeted siRNA Cream in Mouse Skin Tissue

**FIGURE 4:**
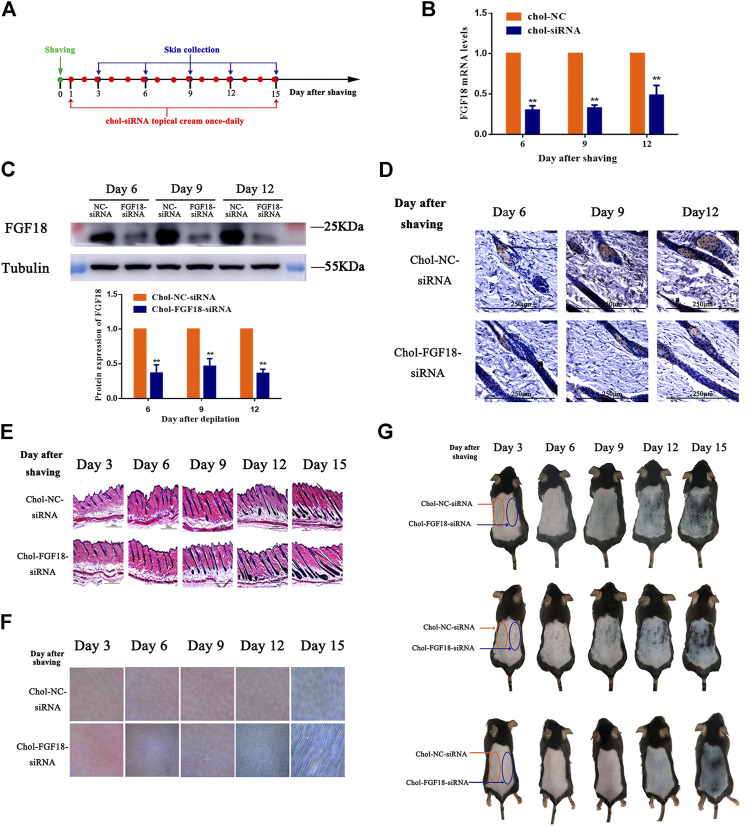
Effects of topical application of FGF18-targeted siRNA cream on hair growth on mouse skin following depilation **(A)** Diagram depicting the timeline in the establishment of the model used for the topical application of Chol-FGF18-siRNA containing cream and collection of skin samples. A cream containing Chol-FGF18-siRNA or Chol-NC-siRNA was applied onto the skin one day after depilation, and changes in the mRNA level of FGF18 in the skin tissue were measured by qPCR **(B)** whereas changes in the protein level of FGF18 were measured by western blot **(C)** at the indicated times. The plot below the blot in **(C)** shows the quantitation of the protein bands in the blot as determined by the gray scale. Data are mean ± SD (*n* = 3). **p < 0.01 vs. Chol-NC siRNA **(D)** Immunohistochemical staining of the skin tissue. **(E)** HE staining of skin tissue showing the different phases of the hair follicle cycle **(F)** Representative images showing the extent of hair growth on the skin as detected by a hair follicle detector at 200× magnification. **(G)** Representative images showing the back of the mice after depilation and administration of FGF18-targeted siRNA and NC-siRNA.

Figure 4A shows the timeline for the depilation of mice skin, the topical application of FGF18 siRNA and collection of skin samples for further analysis to assess the impact on the hair follicles. Topical application with a cholesterol-modified FGF18 siRNA cream resulted in significantly lower level of FGF18 mRNA compared with the application of a cholesterol-modified negative control siRNA cream (Figure 4B). Similarly, western blot also revealed a significant decrease in the level of FGF18 protein in skin that was applied with the FGF18 siRNA cream. (Figure 4C). Immunohistochemical analysis of the skin sample confirmed that topical application of FGF18 siRNA could effectively inhibit the expression of FGF18 in the skin, resulting in significantly lower level of FGF5 on days 6, 9 and 12 compared with the application of the negative control siRNA (Figure 4D). On day 9, the hair follicles of the skin applied with the FGF18 siRNA cream exhibited hair growth and plump papillae that were located at the dermal-subcutaneous junction, indicating that the hair follicles had entered the anagen phase. As for the skin treated with the negative control siRNA, similar levels of hair growth were only achieved on day 12 (Figure 4E), demonstrating that the telogen-to-anagen transition occurred significantly earlier when cholesterol-modified siRNA was topically applied onto the skin. Digital images taken of the skin zone where FGF18 siRNA was applied revealed the presence of black spots on day 9, followed by obvious hair growth on day 12 and patches of thick hairs by day 15. In contrast, the zone applied with the negative control siRNA remained pink on day 9 and exhibited little hair growth on day 12. Significant hair growth only appeared on day 15 (Figures 4F,G). These results showed that the topical application of a cholesterol-modified FGF18 siRNA-containing cream could produce effects comparable to those of an intradermal injection. FGF18 gene expression in skin tissue was effectively suppressed, and as a result, the time taken to reach the telogen-to-anagen transition was shortened by 3 days, effectively promoting hair growth.

## Discussion

Hair follicle cycle abnormalities constitute an important pathological feature and a key pathogenic mechanism of alopecia. The key to preventing alopecia is the prevention of the progressive shortening and premature termination of the anagen phase of the hair follicles, and the key to hair growth is the triggering of entry into the anagen phase. Presently, there is abundant research evidence showing that FGF5 is the most crucial regulatory factor that promotes the anagen-to-catagen transition of the hair follicles. As abnormally thick and long hair has been observed in FGF5-deficient animals, FGF5 has become the main target of research seeking to improve the length and quality of the hair coat in animals. Besides prolonging the anagen phase of the hair follicles to promote continuous hair growth, the mobilization of hair follicles in the telogen phase toward the anagen phase is also a key aspect of alopecia treatment. It is worth noting that FGF18 is a key regulatory factor in the maintenance of the telogen phase of hair follicles. Therefore, targeting the regulation of FGF5 and FGF18 could be a potential strategy for preventing hair loss and promoting hair growth.

Finasteride and minoxidil have been approved to use in clinic to promote the hair growth. Finasteride is a dihydrotestosteron-suppressing 5 α-reductase inhibitor, it stimulates hair growth by decreasing the serum levels of dihydrotestosterone. Minoxidil is a vasodilator, it slows or stops hair loss and promotes hair growth via increaseing the cutaneous blood flow to the scalp, allowing more oxygen, blood and nutrients to reach the follicle. Different from the mechanism of minoxidil and finasteride, the objective of this study is use cholesterol-modified siRNA to promote hair growth through changing the hair follicle cycle. In the present study, we inhibited the expression of the FGF5 gene in the skin tissue on the back of mice using a cholesterol-modified FGF5-targeting siRNA. This modified siRNA prolonged the anagen phase of the hair follicles and delayed the anagen-to-catagen transition by 2 days, suggesting that hair follicles are extremely sensitive to changes in the expression of FGF5, as the extension of the anagen phase could be effectively achieved by partially inhibiting FGF5 expression, eliminating the need to perform complex gene editing at the DNA level or targeted gene inactivation. This observation is consistent with the findings reported by Burg (Burg et al., 2017), who found that certain traditional Chinese medicine (TCM) extracts such as *Ginkgo biloba* leaf extract, *Eriobotrya japonica* leaf extract, and *Sanguisorba officinalis* extract can specifically inhibit the activity of FGF5 and prevent hair loss and enhance hair growth. On this basis, hair care products containing FGF5-inhibiting *Ginkgo biloba* leaf extract and *Eriobotrya japonica* leaf extract have been developed by these researchers. Besides, a number of monoterpenoids with FGF5 inhibitory activity were found in the aforementioned TCM extracts, and these monoterpenoids can also prevent hair loss and enhance hair growth in a clinical study. However, the siRNA-mediated inhibition of mRNA expression that we observed was quite effective and more specific compared with the TCM extracts. Taken together, the findings from previous research and our experimental observations could suggest that the use of an FGF5-targeted siRNA may be an optimum method for prolonging the anagen phase of hair follicles and promoting hair regrowth.

FGF18-targeted siRNA also effectively suppressed the expression of the FGF18 gene *in vivo*. Consequently, the telogen-to-anagen transition was shortened by 3 days, suggesting that targeting FGF18 could mobilize the hair follicles in the telogen phase to enter the anagen phase. Considerable research evidence has shown that FGF18 is not the only factor responsible for maintaining the telogen phase of the hair follicles, but that it also interacts with bone morphogenetic protein (BMP) and Sost signaling to maintain the quiescent state of the hair follicles (Kimura-Ueki et al., 2012). Interestingly, although FGF18 and BMPs are key regulatory factors for maintaining hair follicles in the telogen phase and are overexpressed during the refractory telogen, both factors seem to exert their effects independently of each other. Our data have also confirmed this conjecture, i.e., precocious anagen initiation could also be induced by merely blocking FGF18 signaling (Figure 3). However, the possibility that the simultaneous inhibition of FGF18 and BMPs might result in synergistic or additive effects cannot be ruled out.

The cholesterol-modified siRNAs were found to have better activity and stability compared with the CPP-bound siRNAs in both the *in vivo* and *in vitro* experiments. Therefore, in subsequent animal experiments that investigated the regulation of the hair follicle cycle by siRNAs, we selected the cholesterol-modified siRNA as the sole means for the delivery of the siRNA. Our results indicated that the expression of FGF5 or FGF18 remained inhibited for 24–72 h, with more than 50% inhibition after a single injection of the siRNA. Prolonged inhibition of FGF5 or FGF18 expression mediated by multiple injections of the corresponding cholesterol-modified siRNA appeared to prolong the anagen phase of the hair follicles and hastened the entry of the hair follicles in the telogen phase into the anagen phase.

When siRNA first entered clinical trials, the method of administration was intradermal injection. However, intradermal injection was found to cause intolerable pain and result in low siRNA penetration efficiency (Hickerson et al., 2011). To investigate the convenience and effectiveness of minimally invasive and noninvasive siRNA delivery methods, we compared the *in vivo* pharmacological effects of the cholesterol-modified FGF18 siRNA, administered either by intradermal injection or by topical application on the hair follicle cycle in mice. In the depilated mouse model established using seven-week-old C57BL/6 mice, the hair follicles were in the telogen phase, implying the presence of a high level of FGF18 expression in the skin. Therefore, the application of siRNA was performed on the day after depilation, and the inhibitory effect of the FGF18 siRNA was assessed at the appropriate time points. During the telogen phase of the hair follicles, the melanocytic stem cells in the skin were also in a quiescent state, giving rise to a pink appearance in the skin on the back of the depilated mice (Figure 4G). Upon entry into the anagen phase, the melanocytic stem cells were simultaneously activated, which were manifested as the appearance of black patches in the skin of the depilated regions. Therefore, the anagen-inducing effect of the administered siRNA could be determined from the time elapsed between the injection of siRNA and the appearance of these black patches in the depilated skin as well as by the observation of hair growth after depilation. In contrast, to prolong the anagen phase via targeting FGF5, it was necessary to inhibit the expression of FGF5 at the late anagen phase. In the depilation mouse model that we used, the hair follicles in the back skin were able to enter the anagen phase within 24 h after depilation. Consequently, drug administration could only be initiated during the late anagen phase, i.e., Day 14 after depilation, and the mice needed to be observed for a certain period after the late anagen phase (around Day 27; a normal anagen phase lasts approximately 21 days) to determine if the anagen phase had been prolonged by the administered drug. However, the hair coat of the animal became extremely thick by the late anagen phase, making it difficult to determine the end of the anagen phase by naked-eye observations or by the aid of dermatoscope. Thus, it was difficult to observe the regulation of the hair follicles following the disruption of FGF5 expression than the disruption of FGF18 expression. Therefore, we only tried the topical cream experiment for FGF18 siRNA.

Considerable evidence has shown that lipophilic liposomes and cream formulations are capable of delivering large molecules to the hair follicles, which promote transdermal delivery in conjunction with sebaceous glands and facilitate the distribution of macromolecules within the dermis (Dokka et al., 2005)^-^(Colombo et al., 2019). Previous research has reported that the topical application of an emulsified siRNA on the skin surface can lead to the efficient suppression of the target gene in the epidermis, and this can be further improved by the addition of a penetration-enhancing agent. Therefore, we designed a cholesterol-modified siRNA-containing lipophilic cream that can be topically applied to the skin using azone as the penetration-enhancing agent. The inhibition of FGF18 expression exerted by the topical application of the FGF18 siRNA specific cream was comparable with that exerted by the intradermal injection of the same siRNA. Both were able to reduce the time required for the telogen-to-anagen transition by 3 days, suggesting that differences in the formulation of the cream and the relative number and functional integrity of the hair follicles play a key role in the success of this delivery method. Compared with intradermal injections, topical medications possess the key advantages of being noninvasive, less irritating to the skin, and safer to use. The findings from previous studies and those of our experiments jointly demonstrate that the topical application of a cholesterol-modified siRNA-containing cream could potentially be used as a long-term, convenient, and economical method of siRNA administration for regulating target genes in the skin that associated with skin conditions such as alopecia. As the present study is a proof-of-concept study, further research is required to validate the effects of the cholesterol-modified siRNA-containing lipophilic cream in humans, especially in alopecia patients.

## Conclusion

Cholesterol modification of siRNAs targeting FGF5 or FGF18 could facilitate their efficient entry into skin cells and subsequent inhibition of FGF5 or FGF18 expression. Intradermal injections of a cholesterol-modified FGF5 siRNA effectively prolonged the anagen phase of the hair follicles, while intradermal injections of a cholesterol-modified FGF18 siRNA or direct topical application of a cream containing the cholesterol-modified FGF18 siRNA to the skin was found to effectively inhibit the expression of FGF18, thereby promoting the telogen-to-anagen transition by hair follicles. Thus, the prevention of hair loss and promotion of hair regrowth could be achieved through the injection of cholesterol-modified siRNA or topical application of a cholesterol-modified siRNA-containing cream that would inhibit the expression of FGF5 or FGF18 expression. Both types of siRNA formulations could, therefore, be used in the development of novel treatments for alopecia as well as for production of hair growth products.

## Data Availability

The raw data supporting the conclusions of this article will be made available by the authors, without undue reservation, to any qualified researcher
